# P02.166. Group yoga intervention leads to improved balance and balance self-efficacy after stroke

**DOI:** 10.1186/1472-6882-12-S1-P222

**Published:** 2012-06-12

**Authors:** A Schmid, M Van Puymbroeck, K Miller, N Schalk

**Affiliations:** 1 Roudebush VAMC and Indiana University, Indianapolis, USA; 2 Indiana University, Bloomington, USA; 3 Heartland Yoga, Indianapolis, USA

## Purpose

Evaluate the impact of an 8-week group yoga intervention on balance, balance self-efficacy, and falls-efficacy in individuals with chronic stroke.

## Methods

This is a prospective pilot study of a modified yoga intervention. All study participants: had chronic stroke (>9 months); completed all rehabilitation after stroke; were able to stand but self-reported some residual disability related to walking, balance, or functional loss after stroke; and scored > 4 out of 6 on the Short Mini Mental Status Exam. Forty-seven individuals with stroke were recruited and randomized 3:1 to yoga or waitlist control. The yoga group completed one hour yoga sessions twice a week for 8 weeks. Yoga was taught by a certified yoga therapist and included modified physical postures, yoga breathing, bilateral movements, and concluded with relaxation while seated, standing, and supine. Assessments before and after the 8 weeks included: Berg Balance Score (balance), Activities Balance Confidence Scale (ABC, balance self-efficacy), and Falls-Efficacy Scale (falls-efficacy). We compared groups with a t-test/Mann Whitney. We used paired t-tests to compare baseline and 8-week data.

## Results

The average age of participants completing the study was 64; 76% were male; and 63% were white. There were no differences in demographics or outcomes between the yoga and control groups. There were no improvements in the waitlist control group. In the yoga group (n=29), significant improvements were found after the 8-week intervention in balance (Berg 40.7±12.1 vs 47±9.6, p<0.001) and balance self-efficacy (ABC 61.25±21.8 vs 67.2±23.1, p=0.035). Falls-efficacy did not improve (p=0.164).

## Conclusion

Our findings suggest an 8-week yoga intervention impacts balance and balance self-efficacy for people with chronic stroke. Yoga activities may have improved neuromuscular control, allowing for strength improvements in affected limbs/side or areas of disuse, thereby improving balance. Continued testing with a larger sample is warranted to determine the impact of yoga on balance and self-efficacy.

